# Supplementary material for: Effect of SARS-CoV-2 infection on host competing endogenous RNA and miRNA network
Source: PeerJ. 2021 Oct 20;9:e12370. doi: 10.7717/peerj.12370 (PMC8541317; doi:10.7717/peerj.12370)
Supplement: Supplemental Information 2 — Figure S1: General workflow of determining SARS-CoV-2 viral RNA effect on host miRNA:ceRNA interactions. Figure S2 : Differential miRNA expression analysis of Mock cells at 4h versus 24 h. Figure S3 : Correlation of number of cells in which node is perturbing: SARS-CoV-1 versus Mock with 0.96 correlation coefficient Figure S4 : Correlation of number of cells in which node is perturbing: SARS-CoV-2 versus Mock with 0.91 correlation coefficient Table S1 : Common gene nodes which have altered perturbation ability in SARS-CoV-1 and SARS-CoV-2 simulations. Table S2 : Common miRNA nodes which have altered perturbation ability in SARS-CoV-1 and SARS-CoV-2 simulations. [file peerj-09-12370-s002.pdf]

Supplementary Figures and Tables

Selcen Ari Alper Yilmaz

2021-09-27

Contents

Supplementary Figures **2**

Supplementary Tables **5**

REFERENCES **6**

Supplementary Figures

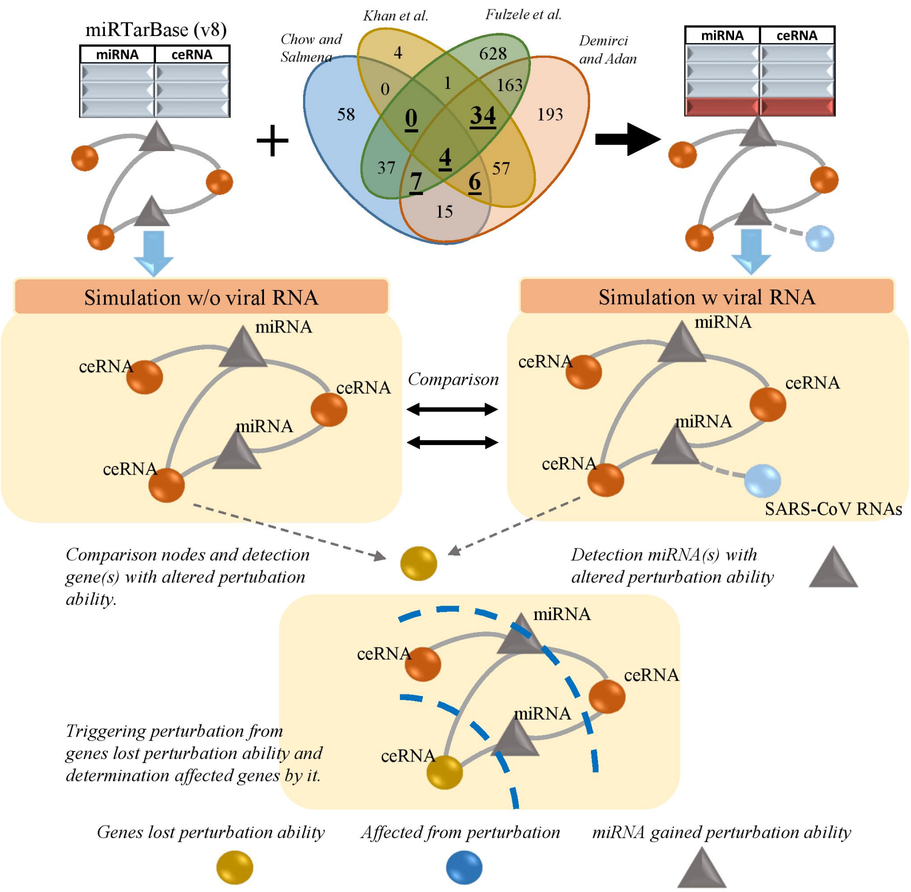

Figure S1: General workflow of determining SARS-CoV-2 viral RNA effect on host miRNA:ceRNA interactions.

General workflow of determining SARS-CoV-2 viral RNA effect on host miRNA:ceRNA interactions. miRTarBase-derived miRNA:target interaction network overlapped with mRNA and miRNA RNA-seq data of each cell. SARS-CoV-2 RNA-targeting miRNAs (underlined-bold) predicted in at least three of the four studies were added to the miRTarBase (v8) data to understand SARS-CoV-2 effect on the host miRNA:ceRNA network (Demirci and Adan 2020; Chow and Salmena 2020; Fulzele et al. 2020; Khan et al. 2020). We performed simulations after construction of network. For SARS-CoV-1, miRNA:SARS-CoV-1 pairs (suggested by (Khan et al. 2020)) were used and the same analyzes were performed. Results from SARS-CoV-1 and SARS-CoV-2 were compared.

Cell-specific simulations were performed for genes that lost their perturbation efficiency in presence of SARS-CoV-2, and genes that were jointly affected (18 affected genes) were selected from the simulations triggered from different genes. The enrichment analysis were performed on miRNA and gene nodes with altered perturbation ability. Also, genes most affected by perturbations were collected and subjected to additonal functional analysis.

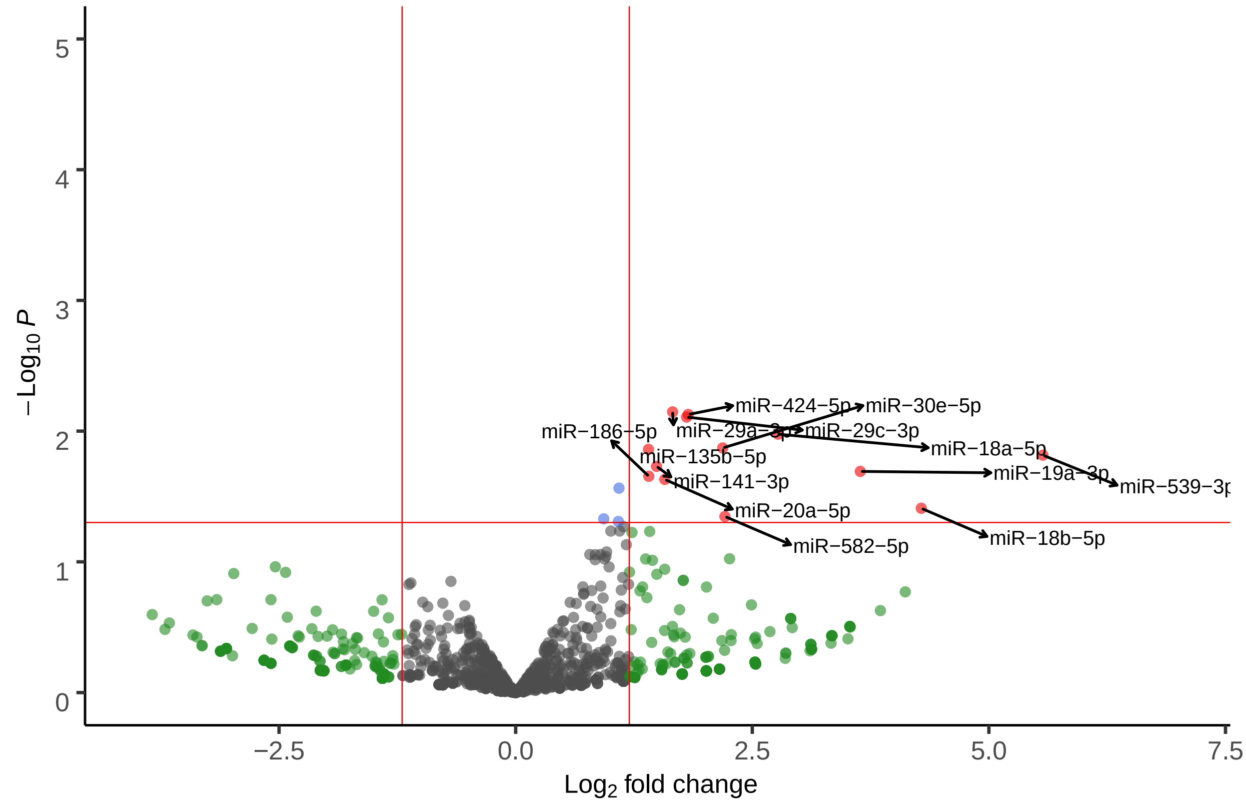

Figure S2: Differential miRNA expression analysis of Mock cells at 4h versus 24h. 13 miRNAs had expressed differentially in mock cells between 4 and 12 h (p-value < 0.05 and $|\log2(FC)| > 1.2$, cut-off with red lines).

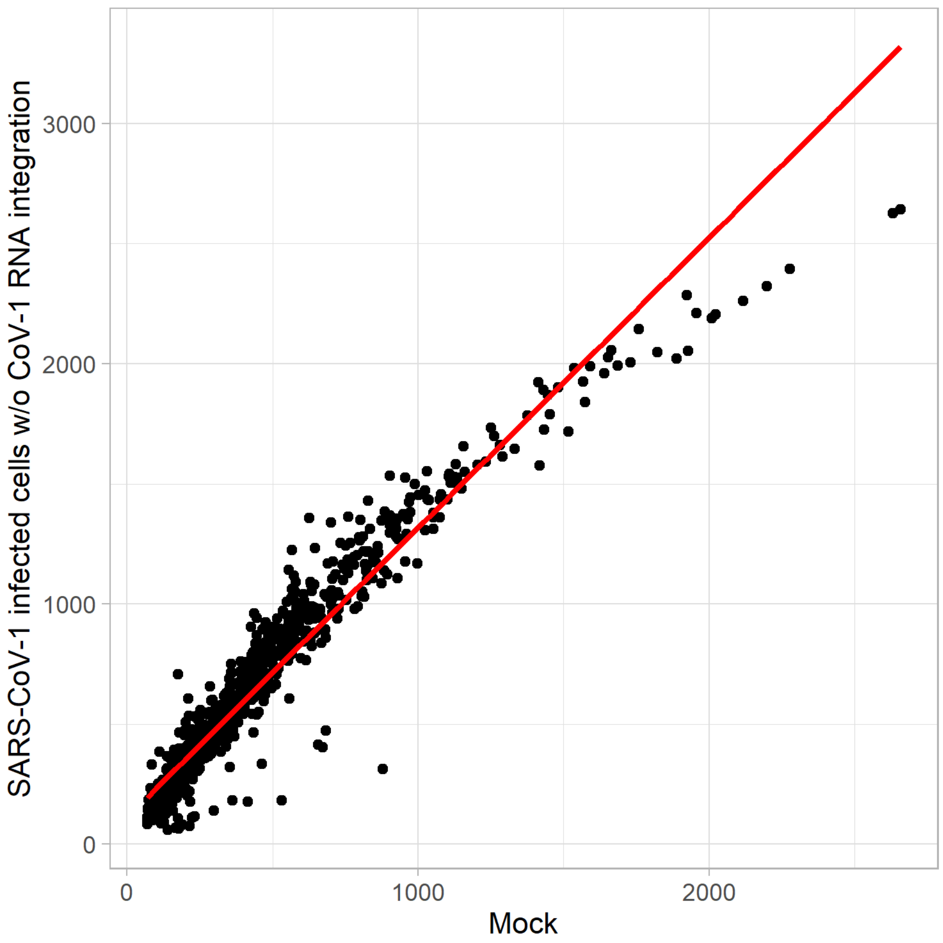

Figure S3: Correlation of number of cells in which node is perturbing: SARS-CoV-1 versus Mock with 0.96 correlation coefficient

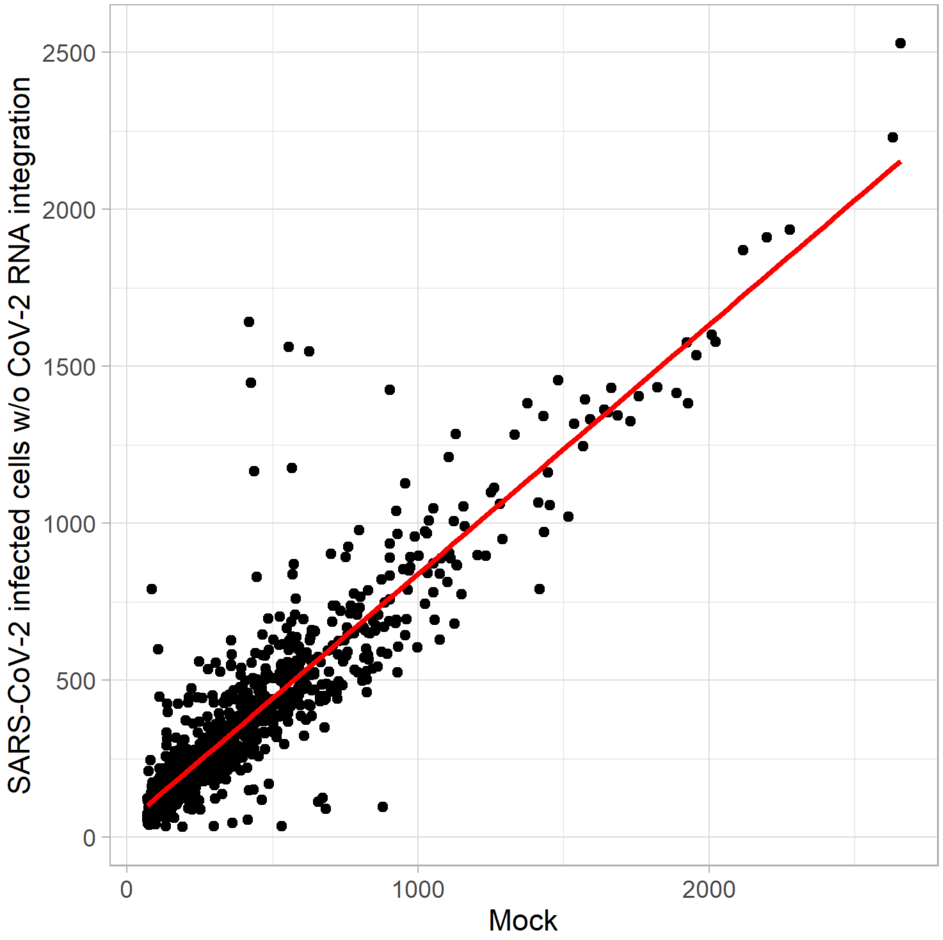

Figure S4: Correlation of number of cells in which node is perturbing: SARS-CoV-2 versus Mock with 0.91 correlation coefficient

Supplementary Tables

Table S1: Common gene nodes which have altered perturbation ability in SARS-CoV-1 and SARS-CoV-2 simulations.

Gene	No of Cells node is pertubing in SARS-CoV-2 simulations	Status in SARS-CoV-2	No of Cells node is pertubing in SARS-CoV-1 simulations	Status in SARS-CoV-1
DIRAS3	64	lost perturbation ability	53	lost perturbation ability

Table S2: Common miRNA nodes which have altered perturbation ability in SARS-CoV-1 and SARS-CoV-2 simulations.

miRNA	No of Cells node is pertubing in SARS-CoV-2 simulations	Status in SARS-CoV-2	No of Cells node is pertubing in SARS-CoV-1 simulations	Status in SARS-CoV-1
hsa-miR-22-5p	143	gained perturbation ability	301	gained perturbation ability
hsa-miR-30c-1-3p	119	gained perturbation ability	2531	gained perturbation ability

REFERENCES

Chow, Jonathan Tak-Sum, and Leonardo Salmena. 2020. "Prediction and Analysis of Sars-Cov-2-Targeting Microrna in Human Lung Epithelium." *Genes* 11 (9): 1002.

Demirci, Müşerref Duygu Saçar, and Aysun Adan. 2020. "Computational Analysis of microRNA-Mediated Interactions in Sars-Cov-2 Infection." *PeerJ* 8: e9369.

Fulzele, Sadanand, Bikash Sahay, Ibrahim Yusufu, Tae Jin Lee, Ashok Sharma, Ravindra Kolhe, and Carlos M Isales. 2020. "COVID-19 Virulence in Aged Patients Might Be Impacted by the Host Cellular Micrornas Abundance/Profile." *Aging and Disease* 11 (3): 509.

Khan, Md, Md Sany, Rabi Us, Md Islam, Abul Bashar Mir Md Islam, and others. 2020. "Epigenetic Regulator miRNA Pattern Differences Among Sars-Cov, Sars-Cov-2, and Sars-Cov-2 World-Wide Isolates Delineated the Mystery Behind the Epic Pathogenicity and Distinct Clinical Characteristics of Pandemic Covid-19." *Frontiers in Genetics* 11: 765.
